# Increased Expression of Rififylin in *A* < 330 Kb Congenic Strain is Linked to Impaired Endosomal Recycling in Proximal Tubules

**DOI:** 10.3389/fgene.2012.00138

**Published:** 2012-08-08

**Authors:** Kathirvel Gopalakrishnan, Sivarajan Kumarasamy, Yanling Yan, Jiang Liu, Andrea Kalinoski, Anbarasi Kothandapani, Phyllis Farms, Bina Joe

**Affiliations:** ^1^Center for Hypertension and Personalized Medicine, University of Toledo College of Medicine and Life SciencesToledo, OH, USA; ^2^Department of Physiology and Pharmacology, University of Toledo College of Medicine and Life SciencesToledo, OH, USA; ^3^Department of Medicine, University of Toledo College of Medicine and Life SciencesToledo, OH, USA; ^4^Institute of Biomedical Engineering, Yanshan UniversityQinhuangdao, China; ^5^Department of Surgery, University of Toledo College of Medicine and Life SciencesToledo, OH, USA; ^6^Advanced Microscopy Imaging Center, University of Toledo College of Medicine and Life SciencesToledo, OH, USA; ^7^Department of Biochemistry and Cancer Biology, University of Toledo College of Medicine and Life SciencesToledo, OH, USA

**Keywords:** carp-2, kidney disease, hypertension, rat, linkage mapping, gene, rffl, proteinuria

## Abstract

Cell surface proteins are internalized into the cell through endocytosis and either degraded within lysosomes or recycled back to the plasma membrane. While perturbations in endosomal internalization are known to modulate renal function, it is not known whether similar alterations in recycling affect renal function. Rififylin is a known regulator of endocytic recycling with E3 ubiquitin protein ligase activity. In this study, using two genetically similar strains, the Dahl Salt-sensitive rat and an S.LEW congenic strain, which had allelic variants within *a* < 330 kb segment containing rififylin, we tested the hypothesis that alterations in endosomal recycling affect renal function. The congenic strain had 1.59-fold higher renal expression of rififylin. Transcriptome analysis indicated that components of both endocytosis and recycling were upregulated in the congenic strain. Transcription of *Atp1a1* and cell surface content of the protein product of *Atp1a1*, the alpha subunit of Na^+^K^+^ATPase were increased in the proximal tubules from the congenic strain. Because rififylin does not directly regulate endocytosis and it is also a differentially expressed gene within the congenic segment, we reasoned that the observed alterations in the transcriptome of the congenic strain constitute a feedback response to the primary functional alteration of recycling caused by rififylin. To test this, recycling of transferrin was studied in isolated proximal tubules. Recycling was significantly delayed within isolated proximal tubules of the congenic strain, which also had a higher level of polyubiquitinated proteins and proteinuria compared with S. These data provide evidence to suggest that delayed endosomal recycling caused by excess of rififylin indirectly affects endocytosis, enhances intracellular protein polyubiquitination and contributes to proteinuria.

## Introduction

The composition of plasma membranes of virtually all eukaryotic cells is established, maintained, and remodeled by exocytosis, endocytosis, and a process of membrane recycling facilitated by endosomes. Cells are estimated to internalize their cell surface equivalent one to five times per hour (Steinman et al., 1983). This rapid removal of membrane from the cell surface is balanced by endosomal recycling pathways, which return most of the endocytosed proteins and lipids back to the plasma membrane (Maxfield and McGraw, 2004). Thus, a stringent regulation of recycling is essential to maintain the balance between endocytic uptake and recycling pathways. Disruptions in endocytosis and recycling are known to adversely affect diverse cellular processes (Yamamoto et al., 2000; Hryciw et al., 2006; Golachowska et al., 2010; Stendel et al., 2010).

Kidneys reabsorb >95% of all proteins filtered through the glomerular apparatus (Nielsen, 1993). Proteinuria is one of the markers of renal dysfunction. Within the apical membranes of proximal tubule cells in the kidney, an extensive endocytic apparatus plays a key role in the reabsorption and degradation of glomerular-filtered albumin and other proteins (Marshansky et al., 2002) and in the recycling of many functionally important membrane transporters (Brown and Stow, 1996). We hypothesized that any alterations in endosomal recycling disrupts cellular homeostasis and thereby could affect renal function. The current study was designed to test whether altered endosomal recycling facilitated by a congenic segment previously mapped on rat chromosome 10 containing rififylin (Gopalakrishnan et al., 2011) can affect renal molecular and cellular physiology and thereby contribute to the extent of protein excretion in a rat model of cardiovascular and renal disease.

## Materials and Methods

### Animals

All of the animal experiments were conducted in accordance with the National Institutes of Health Guide for the Care and Use of Laboratory Animals and as per approved protocols by the institutional animal care and use review committee of the University of Toledo College of Medicine and Life Sciences. The congenic strain used in the current study was constructed in our laboratory using S and LEW rats. The strain is designated as S.LEW (10) × 12 × 2 × 3 × 5 and the construction of this congenic strain is detailed elsewhere (Gopalakrishnan et al., 2011).

### cDNA analyses

mRNA from kidneys of neonates and 53 days old rats were extracted using TRIzol Reagent (Life Technologies). cDNA was obtained by reverse transcription with SuperScript III (Invitrogen) using an Oligo dT primer. Using genomic sequence data for rat *Rffl* gene available at the Ensembl website^1^, sense (5′CAGCTGAAGGAGATCCTGGC3′) and antisense (5′CCATGCAAATCTTACACAGGTTC3′) primers were designed to amplify exons 4–6 of the *Rffl* transcript by PCR. The resultant cDNA product was confirmed by sequencing using services provided by MWG Biotech Inc. DNA alignments were done using the sequence analysis software *Sequencher* from GeneCodes Corporation. Transcript expression of *Rffl* was analyzed by Real-Time PCR (BioRad) and expression levels relative to *Gapdh* were calculated by the 2^−ΔΔCT^ method (Livak and Schmittgen, 2001).

### Immunoblot analyses

Protein lysates were prepared as described previously (Gopalakrishnan et al., 2011) and subjected to Tricine/SDS-PAGE, transferred to PVDF membrane, incubated with specific primary antibodies followed by secondary antibodies and processed by ECL. Membranes were re-probed with monoclonal anti-Gapdh. The immunoblots were analyzed by densitometric scanning using Image J software. Sources of primary antibodies: Cell Signaling Technology (anti-Gapdh), Abcam (anti-Rffl), the Developmental studies hybridoma bank at the University of Iowa (monoclonal antibody against the Na^+^K^+^ATPase α-1 subunit, clone α6F), Santa Cruz Biotechnology (Donkey anti-rabbit IgG-HRP conjugate).

### Early endosome isolation and western blot analysis of Na^+^K^+^ATPase α1 subunit

Early endosome (EE) fractions (Eea-1 and Rab5 positive) were isolated from renal proximal tubules by sucrose flotation centrifugation as previously described (Liu et al., 2011). The enrichment of EE fractions was assessed by the EE marker Eea-1. Equal amount of total proteins (25 μg) from the EE fraction of each sample was precipitated with trichloroacetic acid for subsequent western blot analysis.

### Whole genome transcriptional profiling

RNA was isolated from the kidneys of concomitantly raised, male, 53-day-old S, and congenic rats (*n* = 6 per group) using TRIzol and purified by RNeasy kit (Qiagen). RNA from two animals was pooled. Three such pooled RNA samples from S and congenic rats were hybridized to Affymetrix Rat Expression Arrays 230 2.0. The arrays were scanned at the Genomics core laboratory of the University of Toledo http://www.utoledo.edu/med/depts/bioinfo/cores/genointro.html. Statistical analyses of the microarray data were performed using the R statistical package (version 2.8.1). The microarray data are in compliance with the Minimum Information About Microarray Experiments and were uploaded into the Gene Expression Omnibus database^2^. Pathway analysis was conducted using Ingenuity Systems Pathway Analysis^3^.

### Isolation and primary culture of rat proximal tubule cells

Primary rat proximal tubule (RPT) cells were isolated from cortices of rat kidneys from S and congenic rats as described previously (Liu et al., 2011).

### Labeling of cell surface Na/K-ATPase by biotinylation

Cell surface biotinylation of Na/K-ATPase in proximal tubule primary cultures was performed as previously described (Liu et al., 2002, 2004, 2011). After surface biotinylation with EZ-Link sulfo-NHS-ss-Biotin (Pierce) and immobilization with ImmunoPure immobilized streptavidin-agarose beads (Pierce), biotinylated proteins were eluted after incubation in a 55°C water bath for 30 min, mixed with an equal volume of 2× Laemmli sample buffer, resolved by 10% SDS-PAGE, and then immunoblotted.

### Transferrin recycling

Transferrin recycling was studied as described previously (Gopalakrishnan et al., 2011). In brief, isolated proximal tubules were maintained at 37°C with 5% CO_2_ and allowed to internalize a fluorescent derivative of transferrin (Alexa^488^-Tf, Molecular Probes) for 90 min at 37°C and washed three times with ice cold PBS. Recycling was induced by warming the cells to 37°C in a serum free medium containing 0.1% BSA and a 100-fold excess of unlabelled holotransferrin (Sigma) and monitored by live imaging using a Leica TCS SP5 laser scanning confocal microscope. Just before monitoring, DRAQ5 was added to visualize the nuclei. Cells were imaged using a 488 and 433 laser line in the *XY* plane with scanning set at 30 s intervals for 30 min. Paired time lapse studies were performed in triplicate using the same gain, offset, and laser power settings to ensure that there were no intensity differences due to the acquisition settings between S and Congenic. Mean fluorescent intensity was measured in Image J at individual time points of the acquired images.

### Polyubiquitinated proteins

Polyubiquitin-modified proteins were isolated from kidneys using the Pierce Ubiquitin Enrichment Kit as per previously published procedures (Gopalakrishnan et al., 2011).

### Urinary protein excretion

Urinary Protein Excretion (UPE) determination was done as previously described (Kumarasamy et al., 2011). Briefly, at 53 days of age, rats fed with low salt (0.3% NaCl) was housed individually in metabolic cages and urine was collected over a 24-h period. Urinalysis was conducted using services provided by the University of Toledo Medical Center. The pyrogallol based QuanTtest Red Total Protein Assay from Quantimetrix (Redondo Beach, CA, USA) was used to determine protein concentrations of the urine samples. A VERSAmax microplate reader from Molecular Devices (Sunnyvale, CA, USA) was used to determine absorbance at 600 nm. Protein concentrations were determined by reading against the absorbance of the QuanTtest human protein standards (25–200 mg/dL). UPE data is presented as mg/mg creatinine over a 24-h period.

### Statistical analyses

All phenotypic data obtained from the two groups (congenic and S rats) were statistically analyzed by Student *t*-test. A *p*-value of <0.05 was considered statistically significant. Statistical analyses of the microarray data were performed with robust multiarray averaging and Benjamini and Hochberg adjustment using the R statistical package (version 2.8.1).

## Results

**Figure 1:**
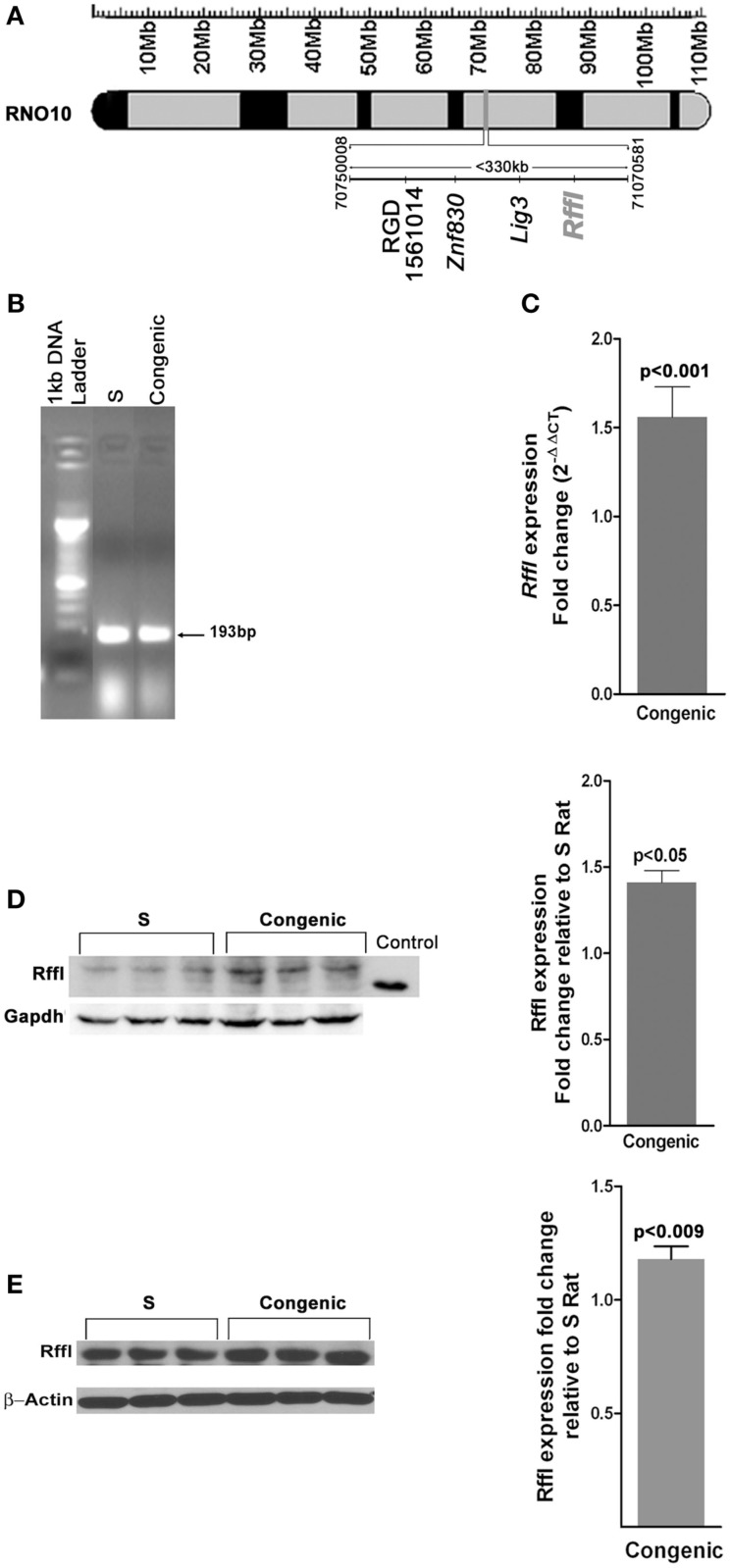
**(A)** Schematic diagram of the congenic strain used in the study. The <330 kb region spanned by the congenic strain S.LEW (10) × 12 × 2 × 3 × 5 is shown alongside the physical map of rat chromosome 10. The basepairs delineating the ends of the congenic segment and the gene annotations were obtained from Ensembl.org. (RGSC 3.4) RNO10, Rat chromosome 10; Mb, Megabases. **(B)** Expression of *Rffl* transcript in the kidneys at 53 days of age as detected by RT-PCR. **(C)** Quantification of *Rffl* transcripts relative to S rats by real-time PCR using whole kidney samples from 53-day-old rats (*n* = 6 animals per group). Immunoblot of Rffl in **(D)** whole-cell lysates from S (*n* = 3) and congenic (*n* = 3) rat kidneys at 53 days of age; **(E)** proximal tubules from S (*n* = 3) and congenic (*n* = 3). RFFL (NP_0010717368, 2aa-99aa, 36.41 kDa) partial recombinant protein was used as positive control and Gapdh was the loading control. Quantification of Rffl protein ± SEM is shown alongside.

The rat strains chosen as tools for this study were the Dahl S rat and *a* > 99% genetically identical strain, the S.LEW congenic strain, which has *a* < 330 kb of the LEW rat genome introgressed onto the genome of the S rat (Figure 1A). At 52 days of age, the systolic blood pressure of the congenic strain measured by the telemetry method was 138 ± 2 mmHg compared with that of the S, 132 ± 2 mmHg, *p* < 0.01 (Gopalakrishnan et al., 2011). The introgressed segment contained the gene rififylin, overexpression of which is known to cause a delay in endosomal recycling in cardiomyocytes (Gopalakrishnan et al., 2011). Rififylin was also transcribed in the kidneys of both the S and the congenic strain (Figure 1B), however, kidneys of congenic rats had a 1.56-fold higher mRNA of rififylin compared with that of the S (*p* < 0.001; Figure 1C). Protein levels of rififylin were also higher both in the kidney and within the proximal tubules of congenic rats compared with S (Figures 1D,E).

**Figure 2:**
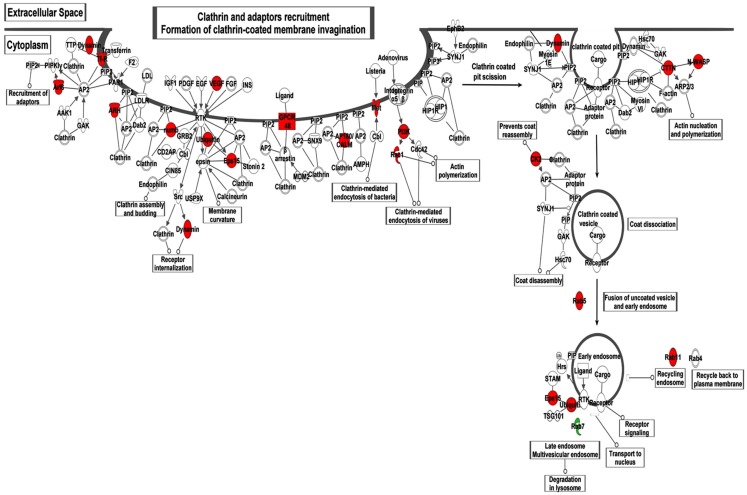
**Illustration of the IPA network analysis of the differentially expressed transcripts associated with Clathrin-mediated endocytosis and recycling**. Transcripts shown in red were upregulated and green were down-regulated in the congenic strain compared with S. The fold changes of the corresponding Affymetrix probes are given in Table 1.

**Figure 3:**
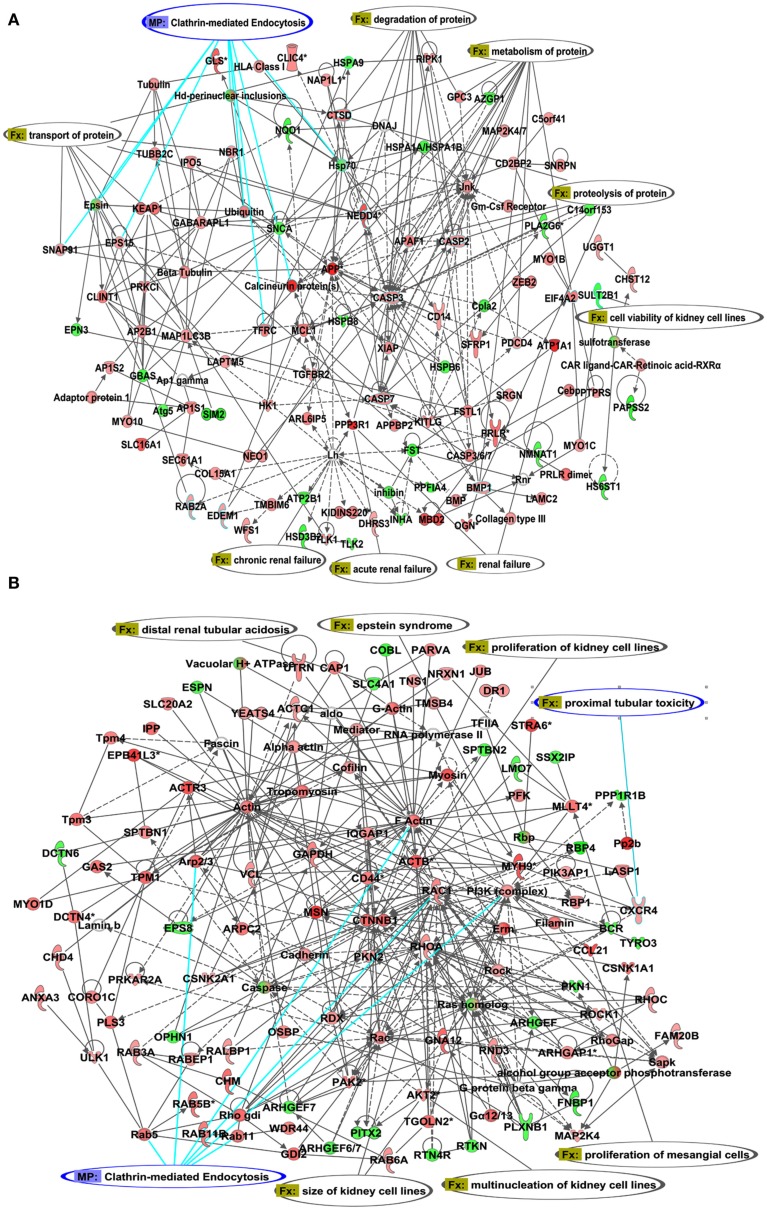
**Illustration of the IPA networks of transcripts associated with cell morphology and renal function**. **(A)** network 1 with *Atp1a1* and **(B)** network with *Rab* proteins Transcripts shown in red were upregulated and transcripts shown in green were down-regulated in the congenic strain compared with S. The fold changes of the corresponding Affymetrix probes are given in Table A1 in Appendix.

To study the alterations in the renal transcriptome between the S and the congenic strain with increased expression of rififylin, a whole genome renal transcriptome analysis was conducted. A total of 1082 probes representing 838 genes and 244 ESTs were upregulated in the congenic strain compared with S. Similarly, a total of 785 probes representing 423 genes and 362 ESTs were down-regulated in the congenic strain compared with S (GSE30770). Among these transcripts, the highest differential expression of 5.33-fold was observed with *Atp1a1*, which was upregulated in the congenic strain compared with S (Table A1 in Appendix). Notably, a number of transcripts coding for proteins either directly or indirectly related to the sorting of endosomes were upregulated in the congenic strain compared with S. The relative changes in gene expression of differentially expressed genes are in Table 1. The networks of these gene products that facilitate clathrin-coated membrane invagination and endocytosis are depicted in Figure 2. The other genes differentially expressed belonged to two prominent networks related to cellular morphology and renal associated function (Figures 3A,B). While *Atp1a1* featured in the network represented in Figure 3A, several transcripts coding for Rab proteins including *Rab5* which regulates transport from plasma membrane to EEs and *Rab11* involved in endocytic recycling (Trischler et al., 1999) featured in the network represented in Figure 3B. The fold changes of all the transcripts within these two additional networks are given in the Table A1 in Appendix.

**Table 1:** **Differentially expressed transcripts in the clathrin-mediated endocytosis network**.

Affymetrix ID	Fold change	*p*-Value	Symbol	Entrez gene name
1369733_at	2.201	0.0258	*Ctnnb1*	Catenin (cadherin-associated protein), beta 1, 88 kDa
1393288_at	1.897	0.0366	*Rab5b*	RAB5B, member RAS oncogene family
1398825_at	1.802	0.0434	*Rab11b*	RAB11B, member RAS oncogene family
1371113_a_at	1.787	0.0411	*Tfrc*	Transferrin receptor (p90, CD71)
1368762_at	1.749	0.0232	*Ubd*	Ubiquitin D
1399153_at	1.715	0.0356	*Rab5b*	RAB5B, member RAS oncogene family
1369998_at	1.708	0.0268	*Arf6*	ADP-ribosylation factor 6
1372513_at	1.63	0.0268	*Rac1*	Ras-related C3 botulinum toxin substrate 1
1388022_a_at	1.459	0.018	*Dnm1l*	Dynamin 1-like
1388104_at	1.436	0.0225	*Igr4*	Leucine-rich repeat containing G protein-coupled receptor 4
1370672_a_at	1.416	0.0422	*Dnm3*	Dynamin 3
1374232_at	1.416	0.0166	*Pik3ca*	Phosphoinositide-3-kinase, catalytic, alpha polypeptide
1384101_at	1.414	0.0362	*Wasl*	Wiskott–Aldrich syndrome-like
1370081_a_at	1.409	0.0236	*Vegfa*	Vascular endothelial growth factor A
1384750_at	1.392	0.037	*Numb*	Numb homolog (*Drosophila*)
1395548_at	1.378	0.0331	*Eps15*	Epidermal growth factor receptor pathway substrate 15
1392643_at	1.355	0.0355	*Rab5b*	RAB5B, member RAS oncogene family
1387170_at	1.238	0.0473	*Csnk2a1*	Casein kinase 2, alpha 1 polypeptide
1368096_at	-1.291	0.0321	*Rab7l1*	RAB7, member RAS oncogene family like 1

Statistical analyses of the microarray data were performed with RMA, robust multiarray averaging; BH, Benjamini and Hochberg adjustment using the R statistical package (version 2.8.1). The complete microarray data is available to the reviewers at the following link: http://www.ncbi.nlm.nih.gov/geo/query/acc.cgi?token=hryjdweamuioidi&acc=GSE30770

**Figure 4:**
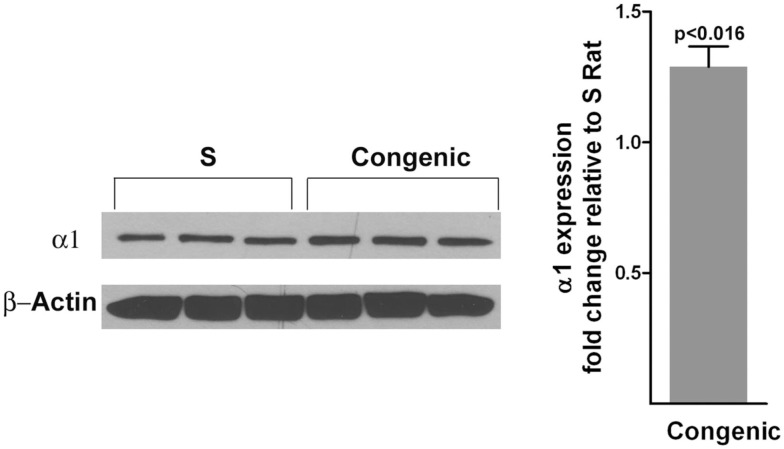
**Quantitation of the α-1 subunit of Na^+^K^+^ATPase on the plasma membranes of cells from proximal tubules (*n* = 3 animals per group)**. The surface biotinylation experiment on isolated proximal tubules was conducted as described under methods. The top panel of the western blot was probed with antibodies to the α-1 subunit of Na^+^K^+^ATPase. The bottom panel was probed with antibodies to β-actin. Densitometric scans are shown on the right hand side.

**Figure 5:**
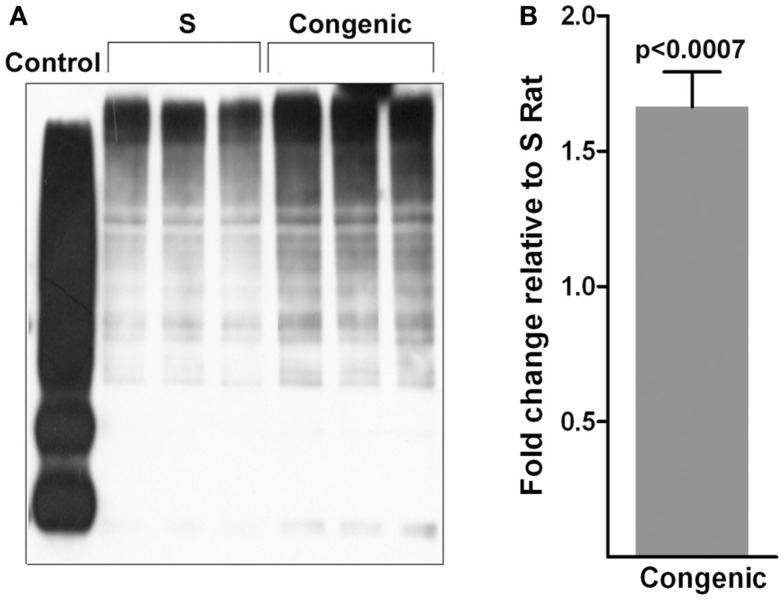
**Polyubiquitination of proteins**. **(A)** Immunoblot of polyubiquitinated proteins in whole-cell lysates from S (*n* = 3) and congenic (*n* = 3) rat kidneys. Control is from the Pierce ubiquitination kit. **(B)** Quantification of the blot shown in **(A)** by densitometry.

Next, we assessed the content of the protein product of the most differentially expressed gene, *Atp1a1*. Within the proximal tubules, the total protein content of the alpha subunit of Na^+^K^+^ATPase (referred to hereafter as alpha 1) was not different between S and the congenic strain (data not shown). Protein levels of alpha 1 were not different between the early endosomal fractions isolated from the proximal tubules of the congenic strain and the S (data not shown). However, surface biotinylation experiments indicated that the content of alpha 1 was notably higher on the cell membranes from the congenic strain compared with S (Figure 4). Total polyubiquitinated proteins were also significantly higher in the congenic strain compared with S (Figure 5).

**Figure 6:**
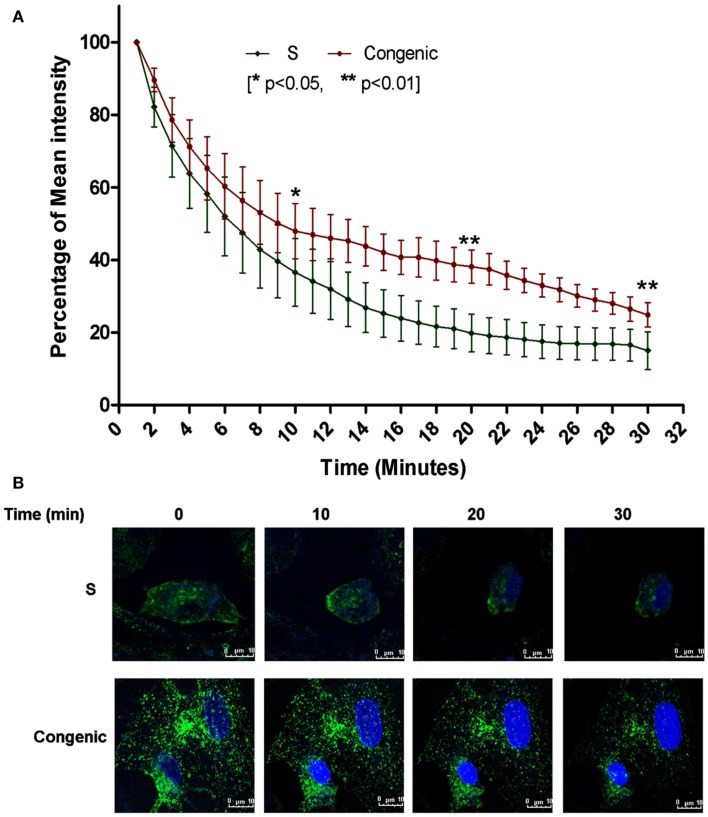
**Defective transferrin recycling in the proximal tubules from the congenic rats**. **(A)** The disappearance of fluorescently labeled transferrin was plotted using the initial mean intensity of labeled internalized transferrin (±SEM) by the proximal tubules taken as 100% in three independent experiments (*n* = 3 animals per group) conducted in duplicates (**p < 0.01; *p < 0.05). **(B)** Representative images, green – fluorescently labeled transferring, blue – DRAQ staining of nuclei.

To assess the extent of endosomal recycling in the kidney of the congenic strain with increased expression of *Rffl*, recycling of fluorescently labeled transferrin was monitored in individual proximal tubules. As shown in Figures 6A,B, recycling of transferrin was significantly delayed in the congenic strain compared with S.

**Figure 7:**
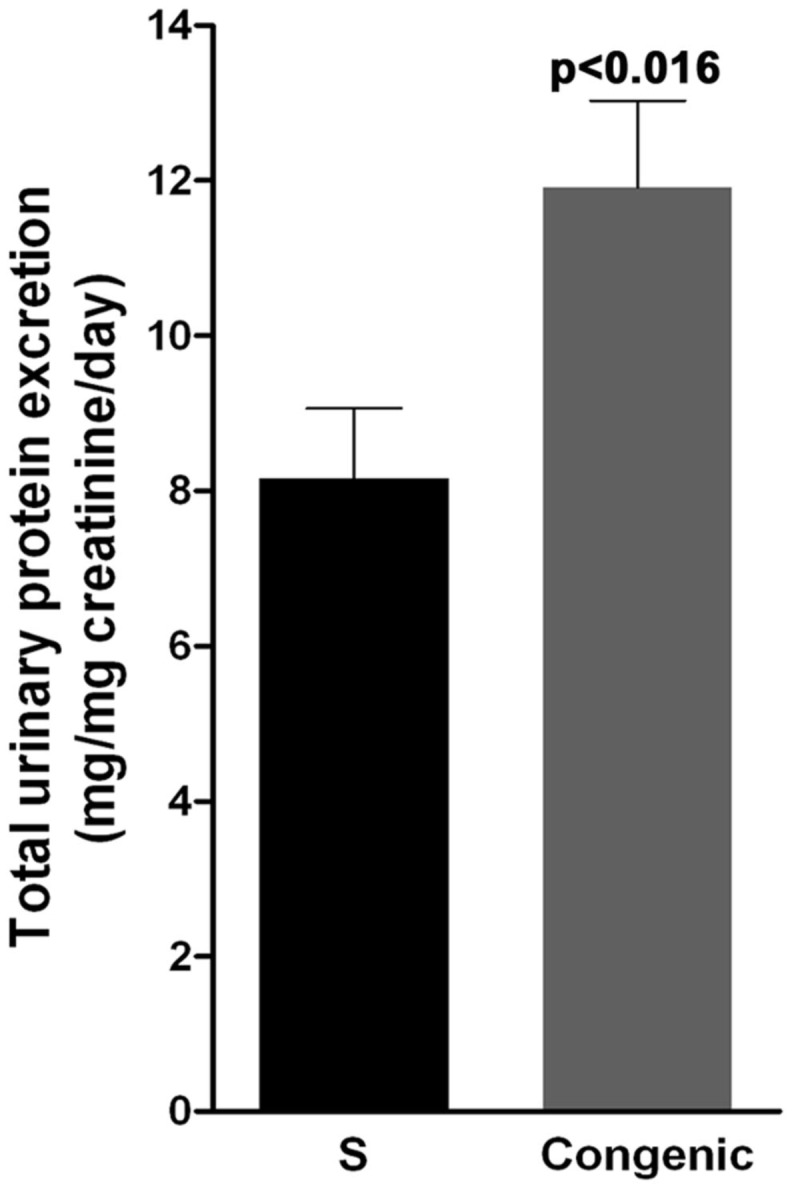
**Urinary protein excretion in S and congenic rats**. Twenty-four hours urine samples were collected from S (*n* = 13) and congenic (*n* = 12) rats and total protein was quantitated. UPE data is presented as mg/mg creatinine over a 24-h period.

These observations, coupled with the fact that rififylin residing within the congenic segment is a regulator of cellular protein recycling, suggested that the primary delay in recycling of endosomes caused membrane proteins to accumulate intracellularly within the proximal tubules from the congenic strain. Because similar defects in membrane traffic and enhanced degradation of proteins are known to cause proteinuria (Marshansky et al., 2002), we tested the urine composition of the two rat strains at a very young age of 53 days. The total protein excretion was significantly higher by 31% in the congenic strain (11.91 ± 1.12 mg/mg creatinine/day) compared to that in the S (8.26 ± 1.08 mg/mg creatinine/day, *p* = 0.016; Figure 7). The other urinary parameters analyzed, i.e., urea nitrogen, glucose, and creatinine excretion were not significantly different between the S and the congenic strain (data not shown).

## Discussion

Hypertension in the Dahl S rat is accompanied with proteinuria (Sustarsic et al., 1981; Sterzel et al., 1988; Garrett et al., 2003). Compared to the S rat, both blood pressure (Gopalakrishnan et al., 2011) and UPE are further increased in the congenic strain reported in the current study. We have previously demonstrated that overexpression of rififylin in the neonatal cardiomyocytes of this congenic strain is linked to short QT-interval and hypertension (Gopalakrishnan et al., 2011). While alterations in QT-interval can contribute to the development of hypertension (Baumert et al., 2011), it does not independently explain the observed increase in UPE of the congenic strain. Because rififylin is also reported to be expressed in other tissues (Coumailleau et al., 2004), we suspected that the fundamental cellular mechanism altered by the overexpression of rififylin could be operational in the kidney wherein rififylin is expressed at higher levels in the congenic strain compared with S. The present study provides evidence to suggest that upregulation of rififylin in the congenic strain compared with S is not limited to the heart, but is also observed at least in one additional organ, the kidney. Functional analysis of rififylin revealed that endocytic recycling is delayed within the proximal tubules. The renal transcriptome signature is reminiscent of perturbations in the endosomal sorting and transport pathways, alterations in which are reported to lead to proteinuria (Nielsen, 1994; Nielsen and Christensen, 2010).

Several structural proteins and GTPase regulators are indispensable for recycling endosomes (Grant and Donaldson, 2009; Schweitzer et al., 2011). Rififylin, also known as Carp-2, is a recent addition to the growing list of proteins associated with the cellular recycling machinery. Coumailleau et al. (2004) described that overexpression of rififylin represents a novel means to inhibit recycling. Using deletion mutants, they demonstrated that the amino-terminal region of rififylin is critical for the recruitment of Rffl to recycling endocytic membranes and for the inhibition of recycling. The current study of delayed recycling in proximal tubules caused by increased renal expression of *Rffl* along with a previous similar report on cardiomyocytes from our group (Gopalakrishnan et al., 2011) represent the first two *in vivo* validations of the *in vitro* studies on HeLa cells reported by Coumailleau et al. (2004).

Transcriptome profiling demonstrates that there are numerous changes in gene transcript levels in the kidneys of S versus the congenic strain. According to the IPA network analysis, genes upregulated were in networks including cellular assembly and organization, cellular function and maintenance and cell morphology, all of which are processes known to involve endocytic recycling (Schweitzer et al., 2011). Two lines of evidence further point to impaired endocytic recycling: (1) upregulation of transcripts in the clathrin-mediated endocytosis and recycling pathways and (2) delayed recycling of transferrin.

Additionally, Coumailleau et al. (2004) have reported that rififylin *per se* does not affect endocytosis. Therefore any alteration in endocytosis is perhaps a representation of the concerted cellular feedback response to the primary defect in recycling in order to maintain cellular homeostasis.

A defect in recycling should either demonstrate an increased accumulation of cargo within the endosomes or trigger degradation of proteins. Evidence from increased polyubiquitinated proteins within the proximal tubules of the congenic strain compared with S point to the latter, i.e., upregulation of the cellular degradation machinery. This is not surprising because rififylin is also a known E3 ubiquitin ligase and we have previously demonstrated similar increased cellular polyubiquitination of proteins within the cardiomyocytes of the congenic strain used in the current study compared with S (Gopalakrishnan et al., 2011). Increased accumulation of polyubiquitination leads to cellular stress, which is known to adversely affect proteinuria (Meyer-Schwesinger et al., 2011). Therefore, it is possible that the increased accumulation of polyubiquitinated proteins in the congenic strain relative to the S, atleast in part, contributes to the observed increased in proteinuria of the congenic strain.

The increase in blood pressure of this strain has been previously attributed partly to increased heart rate observed in the congenic strain (Gopalakrishnan et al., 2011). The current study indicates that an additional factor contributing to the increased blood pressure of the congenic strain could be due to the compensatory mechanism of increased transcription and availability of the Na^+^K^+^ATPase at the surface of cells within the proximal tubules, which may cause increased sodium retention and thereby increase blood pressure.

Overall, three main reasons lead us to conclude that overexpression of rififylin within the congenic strain compared with S is a contributor to the observed alterations in kidney function as noted by alterations in proteinuria – (1) the two strains compared were genetically identical except for the very short <330 kb congenic segment harboring rififylin; (2) two known functional consequences of delayed endocytic recycling and accumulation of polyubiquitinated proteins (Coumailleau et al., 2004, 2005) as a result of overexpression of rififylin were recapitulated in the congenic strain; and (3) *Rffl* is a candidate gene within the congenic interval that is reported to affect both recycling and polyubiquitination. Despite these compelling arguments, it remains to be determined using future mapping studies to further dissect the <330 kb congenic segment as to whether additional factors within the congenic interval also contribute to the reported phenotypes.

Given that alpha1 is not within the congenic segment, it is also reasonable to conclude that the primary physiological perturbations that may have lead to the observed increase in transcription of alpha1 and the increased alpha 1 content on the plasma membrane is a compensatory mechanism. Of course, we would expect increased blood pressure as one of the consequences to this compensatory mechanism and the congenic strain indeed has higher blood pressure at a very young age of 52 days. Further, a prolonged cellular stress as a result of accumulation of excess proteins marked for degradation could be viewed as being highly detrimental because the congenic strain is reported to have a decreased life span compared with S (Gopalakrishnan et al., 2011).

Genome-wide association and linkage studies in humans and model organisms point to a number of candidate genes for chronic renal disease and/or albuminuria (Liu and Freedman, 2005; Krolewski et al., 2006; Turner et al., 2006; Arar et al., 2007, 2008; Garrett et al., 2007, 2010; Hwang et al., 2007; Iyengar et al., 2007; Leon et al., 2007; Martinez et al., 2010; Sterken and Kiryluk, 2010). The genome-wide association studies in particular only represent <1.5% of the total variance in albuminuria observed in human populations. Therefore a large number of loci causing or contributing to renal function disorders in humans remain unidentified. Genome-wide studies have identified single nucleotide polymorphisms around the gene coding for rififylin in humans to QT-intervals (Newton-Cheh et al., 2009; Pfeufer et al., 2009), but not to any renal phenotypes. Through the discovery of a link between endosomal recycling, enhanced degradation, and a resultant altered trafficking of proteins within the proximal tubules, the present study provides the basis for evaluating rififylin as a novel candidate gene for renal disease characterized by proteinuria in humans.

## Conflict of Interest Statement

The authors declare that the research was conducted in the absence of any commercial or financial relationships that could be construed as a potential conflict of interest.

## Footnotes

^1^www.ensembl.org

^2^http://www.ncbi.nlm.nih.gov/geo/query/acc.cgi?token=hryjdweamuioidi&acc=GSE30770

^3^www.ingenuity.com

## Appendix

**Table A1:** **Differentially expressed transcripts in networks (Figures 3A,B)**.

Affymetrix ID	Fold change	*p*-Value	Symbol	Entrez gene name
1371108_a_at	5.3380	0.0334	Atp1a1	ATPase, Na^+^/K^+^ transporting, alpha 1 polypeptide
1380533_at	4.2845	0.0180	App	Amyloid beta (A4) precursor protein
1369152_at	3.3827	0.0319	Ppp3r1	Protein phosphatase 3, regulatory subunit B, alpha
1368948_at	3.1624	0.0383	Msn	Moesin
1370503_s_at	2.9373	0.0422	Epb41l3	Erythrocyte membrane protein band 4.1-like 3
1390525_a_at	2.7414	0.0397	Stra6	Stimulated by retinoic acid gene 6 homolog (mouse)
1378015_at	2.6839	0.0206	Ccl21	Chemokine (C–C motif) ligand 21
1388774_at	2.6075	0.0051	Mbd2	Methyl-cpg binding domain protein 2
1383899_at	2.5886	0.0335	Nedd4	Neural precursor cell expressed, developmentally down-regulated 4
1395886_at	2.5617	0.0327	Actr3	ARP3 actin-related protein 3 homolog (yeast)
1387402_at	2.5213	0.0303	Myh9	Myosin, heavy chain 9, non-muscle
AFFX_Rat_beta-actin_5_at	2.4164	0.0236	Actb	Actin, beta
1386981_at	2.4077	0.0157	Slc16a1	Solute carrier family 16, member 1 (monocarboxylic acid transporter 1)
1382616_at	2.3050	0.0359	Gls	Glutaminase
1369733_at	2.2009	0.0258	Ctnnb1	Catenin (cadherin-associated protein), beta 1, 88 kDa
1370288_a_at	2.1568	0.0201	Tpm1	Tropomyosin 1 (alpha)
1369278_at	2.1499	0.0202	Gna12	Guanine nucleotide binding protein (G protein) alpha 12
1398822_at	2.0847	0.0284	Gdi2	GDP dissociation inhibitor 2
1369227_at	2.0708	0.0137	Chm	Choroideremia (Rab escort protein 1)
1392406_at	2.0290	0.0370	Ipp	Intracisternal A particle-promoted polypeptide
1370789_a_at	2.0256	0.0435	Prlr	Prolactin receptor
1369312_a_at	2.0143	0.0280	Csnk1a1	Casein kinase 1, alpha 1
1371028_at	2.0125	0.0372	Tgoln2	Trans-golgi network protein 2
1371139_at	2.0094	0.0475	Pls3	Plastin 3
1387810_at	2.0016	0.0177	Keap1	Kelch-like ECH-associated protein 1
1396267_at	1.9907	0.0392	Pak2	p21 protein (Cdc42/Rac)-activated kinase 2
1368537_at	1.9863	0.0225	Dctn4	Dynactin 4 (p62)
1383263_at	1.9628	0.0177	Ogn	Osteoglycin
1387392_at	1.9581	0.0276	Mllt4	Myeloid/lymphoid or mixed-lineage leukemia; translocated to, 4
1369779_at	1.9506	0.0099	Myo1d	Myosin ID
1375137_at	1.9173	0.0366	Arpc2	Actin-related protein 2/3 complex, subunit 2, 34 kDa
1379452_at	1.9090	0.0372	Gas2	Growth arrest-specific 2
1371239_s_at	1.9033	0.0379	Tpm3	Tropomyosin 3, gamma
1393288_at	1.8966	0.0366	Rab5b	RAB5B, member RAS oncogene family
1370141_at	1.8715	0.0280	Mcl1	Myeloid cell leukemia sequence 1 (BCL2-related)
1394965_at	1.8507	0.0450	Clint1	Clathrin interactor 1
1387952_a_at	1.8175	0.0221	Cd44	CD44 molecule (Indian blood group)
1398825_at	1.8017	0.0434	Rab11b	RAB11B, member RAS oncogene family
1397697_at	1.7912	0.0321	Eif4a2	Eukaryotic translation initiation factor 4A2
1371113_a_at	1.7874	0.0411	Tfrc	Transferrin receptor (p90, CD71)
1367651_at	1.7835	0.0464	Ctsd	Cathepsin D
1398311_a_at	1.7425	0.0411	Kidins220	Kinase d-interacting substrate, 220 kDa
1369197_at	1.7379	0.0268	Apaf1	Apoptotic peptidase activating factor 1
1368808_at	1.7327	0.0282	Cap1	CAP, adenylate cyclase-associated protein 1 (yeast)
1368838_at	1.7273	0.0388	Tpm4	Tropomyosin 4
1396214_at	1.7272	0.0205	Kitlg	KIT ligand
1369319_at	1.7265	0.0333	Arl6ip5	ADP-ribosylation-like factor 6 interacting protein 5
1388251_at	1.7156	0.0368	Prkci	Protein kinase C, iota
1396072_at	1.6782	0.0364	Appbp2	Amyloid beta precursor protein (cytoplasmic tail) binding protein 2
1382878_at	1.6562	0.0224	Sfrp1	Secreted frizzled-related protein 1
1391543_at	1.6489	0.0266	Ripk1	Receptor (TNFRSF)-interacting serine-threonine kinase 1
1382615_at	1.6466	0.0460	Sec61a1	Sec61 alpha 1 subunit (*S. cerevisiae*)
1397843_at	1.6417	0.0473	Wdr44	WD repeat domain 44
1370662_a_at	1.6351	0.0421	Ap2b1	Adaptor-related protein complex 2, beta 1 subunit
1369720_at	1.6345	0.0257	Myo1b	Myosin IB
1377769_at	1.6329	0.0402	Ap1s1	Adaptor-related protein complex 1, sigma 1 subunit
1372513_at	1.6297	0.0268	Rac1	Ras-related C3 botulinum toxin substrate 1
1387844_at	1.6240	0.0175	Lasp1	LIM and SH3 protein 1
1396250_at	1.6206	0.0311	Coro1c	Coronin, actin binding protein, 1C
1368832_at	1.6121	0.0337	Akt2	v-akt murine thymoma viral oncogene homolog 2
1369557_at	1.6047	0.0093	Casp7	Caspase 7, apoptosis-related cysteine peptidase
1393795_at	1.5994	0.0392	Zeb2	Zinc finger E-box binding homeobox 2
1368821_at	1.5950	0.0180	Fstl1	Follistatin-like 1
1383827_at	1.5947	0.0439	Tlk1	Tousled-like kinase 1
1378816_a_at	1.5832	0.0221	osbp	Oxysterol binding protein
1369234_at	1.5818	0.0282	Slc20a2	Solute carrier family 20 (phosphate transporter), member 2
1368395_at	1.5704	0.0307	Gpc3	Glypican 3
1388230_at	1.5600	0.0327	Jub	Jub, ajuba homolog (*Xenopus laevis*)
1370266_at	1.5554	0.0327	Parva	Parvin, alpha
1395132_at	1.5543	0.0221	Utrn	Utrophin
1367974_at	1.5493	0.0342	Anxa3	Annexin A3
1387420_at	1.5445	0.0202	Clic4	Chloride intracellular channel 4
1387690_at	1.5417	0.0180	Casp3	Caspase 3, apoptosis-related cysteine peptidase
1397200_at	1.5319	0.0277	Chd4	Chromodomain helicase DNA binding protein 4
1390706_at	1.5289	0.0323	Sptbn1	Spectrin, beta, non-erythrocytic 1
AFFX_Rat_Hexokinase_3_at	1.5278	0.0388	Hk1	Hexokinase 1
1370599_a_at	1.5255	0.0292	Ptprs	Protein tyrosine phosphatase, receptor type, S
1388762_at	1.5226	0.0374	Iqgap1	IQ motif containing GTPase activating protein 1
1369248_a_at	1.5212	0.0442	Xiap	X-linked inhibitor of apoptosis
1367939_at	1.5168	0.0362	Rbp1	Retinol binding protein 1, cellular
1384938_at	1.5126	0.0470	Arhgap1	Rho GTPase activating protein 1
1369879_a_at	1.4963	0.0208	Tmbim6	Transmembrane BAX inhibitor motif containing 6
1378287_at	1.4835	0.0421	Rdx	Radixin
1371127_at	1.4768	0.0137	Bmp1	Bone morphogenetic protein 1
1371056_at	1.4595	0.0335	Neo1	Neogenin 1
1378842_at	1.4550	0.0465	Gabarapl1	GABA(A) receptor-associated protein like 1
1379889_at	1.4488	0.0321	Lamc2	Laminin, gamma 2
1367891_a_at	1.4455	0.0175	Casp2	Caspase 2, apoptosis-related cysteine peptidase
1396742_at	1.4423	0.0137	Ipo5	Importin 5
1388557_at	1.4420	0.0404	Tubb2c	Tubulin, beta 2C
1367981_at	1.4338	0.0436	Rabep1	Rabaptin, RAB GTPase binding effector protein 1
1393055_at	1.4328	0.0399	Pkn2	Protein kinase N2
1369085_s_at	1.4298	0.0323	Snrpn	Small nuclear ribonucleoprotein polypeptide N
1371103_at	1.4287	0.0268	Rab6a	RAB6A, member RAS oncogene family
1384005_at	1.4212	0.0355	Dr1	Down-regulator of transcription 1, TBP-binding (negative cofactor 2)
1394077_at	1.4202	0.0337	Rnd3	Rho family GTPase 3
1370130_at	1.4201	0.0393	Rhoa	Ras homolog gene family, member A
1379345_at	1.4034	0.0457	Col15a1	Collagen, type XV, alpha 1
1373473_a_at	1.3975	0.0307	Nap1l1	Nucleosome assembly protein 1-like 1
1375538_at	1.3927	0.0399	Vcl	Vinculin
1384187_at	1.3826	0.0287	Ap1s2	Adaptor-related protein complex 1, sigma 2 subunit
1369816_at	1.3815	0.0369	Rab3a	RAB3A, member RAS oncogene family
1368218_at	1.3797	0.0424	Ralbp1	ralA binding protein 1
1395548_at	1.3777	0.0331	Eps15	Epidermal growth factor receptor pathway substrate 15
1385797_at	1.3757	0.0377	Actc1	Actin, alpha, cardiac muscle 1
1382402_at	1.3687	0.0321	Ulk1	Unc-51-like kinase 1 (*C. elegans*)
1371059_at	1.3669	0.0302	Prkar2a	Protein kinase, cAMP-dependent, regulatory, type II, alpha
1383531_at	1.3571	0.0342	C5orf41	Chromosome 5 open reading frame 41
1381509_at	1.3482	0.0341	Nbr1	Neighbor of BRCA1 gene 1
1383701_at	1.3470	0.0434	Map2k4	Mitogen-activated protein kinase kinase 4
1373865_at	1.3468	0.0436	Snap91	Synaptosomal-associated protein, 91 kda homolog (mouse)
1382199_at	1.3447	0.0327	Map1lc3b	Microtubule-associated protein 1 light chain 3 beta
1387356_at	1.3411	0.0333	Wfs1	Wolfram syndrome 1 (wolframin)
1369653_at	1.3385	0.0373	Tgfbr2	Transforming growth factor, beta receptor II (70/80 kDa)
1371659_at	1.3332	0.0472	Rhoc	Ras homolog gene family, member C
1391390_at	1.3288	0.0236	Tns1	Tensin 1
1368006_at	1.3273	0.0236	Laptm5	Lysosomal protein transmembrane 5
1387654_at	1.3194	0.0428	Myo1c	Myosin IC
1393639_at	1.3177	0.0441	Myo10	Myosin X
1370097_a_at	1.3168	0.0374	Cxcr4	Chemokine (C-X-C motif) receptor 4
1395782_at	1.3153	0.0421	Yeats4	YEATS domain containing 4
1384186_at	1.3133	0.0334	Edem1	ER degradation enhancer, mannosidase alpha-like 1
1370087_at	1.3130	0.0327	Rab2a	RAB2A, member RAS oncogene family
1368953_at	1.3126	0.0437	Uggt1	UDP-glucose glycoprotein glucosyltransferase 1
1368490_at	1.3119	0.0212	Cd14	CD14 molecule
1392174_at	1.2989	0.0270	Chst12	Carbohydrate (chondroitin 4) sulfotransferase 12
1368932_at	1.2889	0.0411	Rock1	Rho-associated, coiled-coil containing protein kinase 1
1387521_at	1.2804	0.0470	Pdcd4	Programmed cell death 4 (neoplastic transformation inhibitor)
1380993_at	1.2497	0.0412	Fam20b	Family with sequence similarity 20, member B
1368655_at	1.2456	0.0321	Srgn	Serglycin
1387170_at	1.2384	0.0473	Csnk2a1	Casein kinase 2, alpha 1 polypeptide
1369404_a_at	1.2274	0.0415	Nrxn1	Neurexin 1
1373240_at	1.2135	0.0381	Dhrs3	Dehydrogenase/reductase (SDR family) member 3
1376795_at	1.2133	0.0406	Pik3ap1	Phosphoinositide-3-kinase adaptor protein 1
1385676_at	1.2077	0.0434	Cd2bp2	CD2 (cytoplasmic tail) binding protein 2
1371762_at	−3.6666	0.0137	Rbp4	Retinol binding protein 4, plasma
1376047_at	−1.6495	0.0180	Papss2	3′-phosphoadenosine 5′-phosphosulfate synthase 2
1385722_at	−1.6489	0.0369	Sim2	Single-minded homolog 2 (*Drosophila*)
1387843_at	−1.5384	0.0180	Fst	Follistatin
1368578_at	−1.4838	0.0370	Hsd3b2	Hydroxy-delta-5-steroid dehydrogenase, 3 beta- and steroid delta-isomerase 2
1372208_at	−1.4789	0.0333	Ppp1r1b	Protein phosphatase 1, regulatory (inhibitor) subunit 1B
1378632_at	−1.4769	0.0303	Ppfia4	Protein tyrosine phosphatase, receptor type, interacting protein, alpha 4
1384834_at	−1.4525	0.0321	Cobl	Cordon-bleu homolog (mouse)
1376175_at	−1.4394	0.0371	Gbas	Glioblastoma amplified sequence
1387599_a_at	−1.4347	0.0175	Nqo1	NAD(P)H dehydrogenase, quinone 1
1388721_at	−1.4340	0.0306	Hspb8	Heat shock 22 kDa protein 8
1377342_s_at	−1.4313	0.0333	Fnbp1	Formin binding protein 1
1376248_at	−1.4056	0.0402	Sult2b1	Sulfotransferase family, cytosolic, 2B, member 1
1368247_at	−1.3986	0.0335	Hspa1a/hspa1b	Heat shock 70 kDa protein 1A
1378069_at	−1.3825	0.0259	Pkn1	Protein kinase N1
1387234_at	−1.3696	0.0321	Azgp1	Alpha-2-glycoprotein 1, zinc-binding
1370385_at	−1.3498	0.0268	Pla2g6	Phospholipase A2, group VI (cytosolic, calcium-independent)
1388972_at	−1.3494	0.0215	Rtn4r	Reticulon 4 receptor
1367953_at	−1.3473	0.0492	Tyro3	TYRO3 protein tyrosine kinase
1372467_at	−1.3411	0.0425	Hs6st1	Heparan sulfate 6-*O*-sulfotransferase 1
1387898_at	−1.3285	0.0234	Hspb6	Heat shock protein, alpha-crystallin-related, B6
1397224_at	−1.3272	0.0287	Atp2b1	ATPase, Ca++ transporting, plasma membrane 1
1395499_at	−1.3089	0.0335	Eps8	Epidermal growth factor receptor pathway substrate 8
1372265_at	−1.3030	0.0459	C14orf153	Chromosome 14 open reading frame 153
1373494_at	−1.2980	0.0259	Bcr	Breakpoint cluster region
1379413_at	−1.2966	0.0378	Nmnat1	Nicotinamide nucleotide adenylyltransferase 1
1367812_at	−1.2966	0.0334	Sptbn2	Spectrin, beta, non-erythrocytic 2
1378198_at	−1.2959	0.0333	Ophn1	Oligophrenin 1
1367977_at	−1.2942	0.0406	Snca	Synuclein, alpha (non-A4 component of amyloid precursor)
1368774_a_at	−1.2666	0.0399	Espn	Espin
1372638_at	−1.2656	0.0434	Arhgef7	Rho guanine nucleotide exchange factor (GEF) 7
1368785_a_at	−1.2595	0.0406	Pitx2	Paired-like homeodomain 2
1382055_at	−1.2588	0.0316	Rtkn	Rhotekin
1387124_at	−1.2526	0.0499	Inha	Inhibin, alpha
1376041_at	−1.2509	0.0321	Epn3	Epsin 3
1396392_at	−1.2474	0.0446	Dctn6	Dynactin 6
1384319_at	−1.2451	0.0436	Tlk2	Tousled-like kinase 2
1373146_at	−1.2379	0.0323	Ssx2ip	Synovial sarcoma, X breakpoint 2 interacting protein
1374444_at	−1.2367	0.0424	Plxnb1	Plexin B1
1391915_at	−1.2313	0.0411	Hspa9	Heat shock 70 kDa protein 9 (mortalin)
1385526_at	−1.2223	0.0441	Atg5	ATG5 autophagy related 5 homolog (*S. cerevisiae*)
1381190_at	−1.2181	0.0444	Lmo7	LIM domain 7
1387656_at	−1.2131	0.0463	Slc4a1	Solute carrier family 4, anion exchanger, member 1

Statistical analyses of the microarray data were performed with RMA, robust multiarray averaging; BH, Benjamini and Hochberg adjustment using the R statistical package (version 2.8.1). The complete microarray data is available to the reviewers at the following link: http://www.ncbi.nlm.nih.gov/geo/query/acc.cgi?token=hryjdweamuioidi&acc=GSE30770
